# Supplementary material for: Alcohol Consumption, High-Density Lipoprotein Particles and Subspecies, and Risk of Cardiovascular Disease: Findings from the PREVEND Prospective Study
Source: Int J Mol Sci. 2024 Feb 14;25(4):2290. doi: 10.3390/ijms25042290 (PMC10889823; doi:10.3390/ijms25042290)
Supplement: Supplementary file 1 [file ijms-25-02290-s001.zip › ijms-2812345-supplementary.pdf]

Supplementary materials

**Table S1**	Linear regression analyses showing relationships of HDL functionality measures with alcohol consumption
**Table S2**	Associations of HDL particles and subspecies with CVD in males and females
**Table S3**	Associations of HDL particles and subspecies with cardiovascular outcomes (with further adjustment for HDL-C)
**Table S4**	Associations of HDL particles and subspecies with cardiovascular outcomes (with further adjustment for HDL-P)
**Table S5**	Associations of HDL particles and subspecies with CVD according to alcohol consumption categories
**Table S6**	STROBE Checklist
**Figure S1**	Derivation of the analytic sample

Table S1. Linear regression analyses showing relationships of HDL functionality measures with alcohol consumption

	**Model 1**		**Model 2**	
**Alcohol consumption, g/day**	**Beta (SE)**	***p*-value**	**Beta (SE)**	***p*-value**
**CETP mass, mg/L**				
0/rarely	ref		ref	
0.1-10	224.2 (182.5)	0.22	230.7 (178.4)	0.20
10-30	-111.3 (202.3)	0.58	-58.3 (198.4)	0.77
>30	-209.2 (232.6)	0.37	-184.3 (238.2)	0.44
**LCAT activity, nmol/mL/hr**				
0/rarely	ref		ref	
0.1-10	2.076 (2.855)	0.47	3.891 (2.784)	0.16
10-30	6.992 (3.150)	0.028	4.053 (2.883)	0.16
>30	8.358 (3.545)	0.020	4.317 (3.178)	0.18
**PLTP activity, AU**				
0/rarely	ref		ref	
0.1-10	0.231 (2.727)	0.93	-0.100 (2.751)	0.97
10-30	0.123 (2.858)	0.97	-0.623 (2.958)	0.83
>30	6.728 (3.725)	0.073	5.899 (3.937)	0.14
**Apolipoprotein A-I, g/L**				
0/rarely	ref		ref	
0.1-10	0.047 (0.065)	0.47	0.052 (0.063)	0.41
10-30	0.112 (0.070)	0.11	0.096 (0.069)	0.17
>30	0.173 (0.077)	0.027	0.153 (0.074)	0.041

Analysis is based on 165 participants

Betas are unstandardized regression coeffi.cients

AU, arbitrary units; CETP, cholesteryl ester transfer protein; CI, confidence interval; LCAT, lecithin:cholesterol acyltransferase; PLTP, phospholipid transfer protein; SE, standard error; ref, denotes the reference category used for comparison

Model 1: Unadjusted

Model 2: Age, sex, smoking status, history of type 2 diabetes, systolic blood pressure, total cholesterol, albumin, gamma glutamyltransferase, and alanine aminotransferase

Table S2. Associations of HDL particles and subspecies with CVD in males and females

HDL exposures Per SD increase	Model 1		Model 2	
	HR (95% CI)	*p*-value	HR (95% CI)	*p*-value
**Males (2445 participants 230 CVD events)**				
HDL-C	0.86 (0.74-1.00)	0.048	0.87 (0.74-1.01)	0.071
HDL-P	0.97 (0.84-1.12)	0.67	0.98 (0.84-1.14)	0.79
Large HDL	0.94 (0.81-1.10)	0.45	0.95 (0.81-1.11)	0.51
Medium HDL	0.86 (0.74-1.00)	0.047	0.87 (0.75-1.01)	0.062
Small HDL	1.11 (0.96-1.28)	0.17	1.11 (0.96-1.28)	0.17
HDL size	0.90 (0.77-1.05)	0.18	0.91 (0.77-1.06)	0.23
H1P	1.09 (0.96-1.24)	0.20	1.08 (0.95-1.24)	0.24
H2P	1.04 (0.89-1.21)	0.63	1.05 (0.90-1.22)	0.56
H3P	0.88 (0.76-1.01)	0.069	0.88 (0.76-1.02)	0.083
H4P	0.94 (0.81-1.09)	0.43	0.95 (0.82-1.10)	0.50
H5P	0.97 (0.85-1.12)	0.72	0.96 (0.83-1.11)	0.59
H6P	0.93 (0.80-1.07)	0.31	0.93 (0.80-1.07)	0.32
H7P	0.98 (0.85-1.14)	0.84	0.99 (0.85-1.16)	0.92
**Females (2706 participants and 93 CVD events)**				
HDL-C	0.79 (0.62-1.01)	0.063	0.81 (0.63-1.05)	0.11
HDL-P	0.77 (0.62-0.96)	0.019	0.79 (0.63-0.99)	0.040
Large HDL	0.90 (0.69-1.17)	0.43	0.92 (0.70-1.20)	0.53
Medium HDL	0.95 (0.76-1.19)	0.67	0.98 (0.78-1.23)	0.85
Small HDL	0.82 (0.65-1.04)	0.098	0.83 (0.66-1.05)	0.12
HDL size	0.90 (0.70-1.17)	0.44	0.92 (0.71-1.20)	0.78
H1P	0.87 (0.71-1.07)	0.19	0.86 (0.70-1.05)	0.14
H2P	0.91 (0.72-1.14)	0.40	0.93 (0.74-1.17)	0.52
H3P	1.03 (0.84-1.26)	0.80	1.05 (0.85-1.29)	0.67
H4P	0.87 (0.69-1.09)	0.22	0.88 (0.70-1.11)	0.29
H5P	0.88 (0.73-1.06)	0.17	0.89 (0.74-1.07)	0.22
H6P	0.94 (0.75-1.18)	0.62	0.95 (0.75-1.19)	0.65
H7P	0.86 (0.71-1.04)	0.11	0.86 (0.71-1.05)	0.15

CI, confidence interval; CVD, cardiovascular disease; H1P-H7P, high-density lipoprotein 1-7 particles (from smallest to largest); HDL-C, high-density lipoprotein cholesterol; HDL-P, high-density lipoprotein particles; HR, hazard ratio; SD, standard deviation

Model 1: Age, smoking status, history of type 2 diabetes, systolic blood pressure, total cholesterol, triglycerides, body mass index, glucose, estimated glomerular filtration rate, high-sensitivity C-reactive protein, albumin, gamma glutamyltransferase, and alanine aminotransferase

Model 2: Model 1 plus alcohol consumption

Table S3. Associations of HDL particles and subspecies with cardiovascular outcomes (with further adjustment for HDL-C)

**HDL exposures Per SD increase**	**Model 1**		**Model 2**	
	**HR (95% CI)**	***p*-value**	**HR (95% CI)**	***p*-value**
**Cardiovascular disease**				
HDL-C	0.82 (0.71-0.95)	0.009	NA	NA
HDL-P	0.90 (0.80-1.02)	0.098	0.99 (0.85-1.16)	0.94
Large HDL	0.92 (0.78-1.08)	0.30	1.17 (0.92-1.47)	0.19
Medium HDL	0.88 (0.78-1.01)	0.070	0.97 (0.82-1.13)	0.66
Small HDL	1.01 (0.89-1.15)	0.88	0.99 (0.87-1.13)	0.92
HDL size	0.89 (0.76-1.04)	0.14	1.09 (0.87-1.37)	0.46
H1P	1.02 (0.91-1.14)	0.74	1.00 (0.90-1.12)	0.94
H2P	0.99 (0.87-1.13)	0.92	0.99 (0.87-1.12)	0.85
H3P	0.92 (0.81-1.04)	0.19	0.98 (0.86-1.11)	0.72
H4P	0.91 (0.80-1.04)	0.19	0.99 (0.85-1.15)	0.87
H5P	0.94 (0.83-1.05)	0.27	0.95 (0.85-1.07)	0.44
H6P	0.93 (0.82-1.06)	0.28	1.00 (0.86-1.16)	0.97
H7P	0.94 (0.83-1.06)	0.30	1.02 (0.88-1.18)	0.83
**Coronary heart disease**				
HDL-C	0.70 (0.58-0.85)	<0.001	NA	NA
HDL-P	0.86 (0.74-0.99)	0.040	1.02 (0.85-1.23)	0.85
Large HDL	0.83 (0.68-1.02)	0.081	1.17 (0.88-1.55)	0.29
Medium HDL	0.82 (0.69-0.96)	0.014	0.94 (0.78-1.14)	0.53
Small HDL	1.04 (0.89-1.21)	0.62	1.02 (0.88-1.19)	0.78
HDL size	0.78 (0.64-0.94)	0.011	1.00 (0.77-1.32)	0.97
H1P	0.99 (0.87-1.13)	0.91	0.97 (0.85-1.10)	0.60
H2P	1.05 (0.91-1.23)	0.49	1.06 (0.91-1.23)	0.44
H3P	0.89 (0.77-1.03)	0.12	0.99 (0.85-1.16)	0.89
H4P	0.81 (0.68-0.97)	0.021	0.91 (0.75-1.10)	0.33
H5P	0.96 (0.84-1.11)	0.59	0.99 (0.86-1.14)	0.87
H6P	0.91 (0.79-1.06)	0.24	1.03 (0.87-1.23)	0.72
H7P	0.88 (0.77-1.02)	0.095	1.01 (0.85-1.19)	0.95
**Stroke**				
HDL-C	1.22 (0.97-1.54)	0.094	NA	NA
HDL-P	1.11 (0.88-1.39)	0.39	0.97 (0.73-1.30)	0.84
Large HDL	1.21 (0.94-1.55)	0.14	1.06 (0.70-1.59)	0.79
Medium HDL	1.18 (0.94-1.48)	0.16	1.08 (0.82-1.44)	0.57
Small HDL	0.90 (0.70-1.15)	0.38	0.92 (0.72-1.19)	0.54
HDL size	1.27 (0.98-1.65)	0.071	1.19 (0.78-1.82)	0.42
H1P	0.97 (0.78-1.21)	0.80	0.99 (0.80-1.23)	0.92
H2P	0.91 (0.71-1.15)	0.43	0.93 (0.73-1.18)	0.53
H3P	1.14 (0.91-1.42)	0.26	1.07 (0.84-1.36)	0.57
H4P	1.10 (0.88-1.37)	0.41	1.00 (0.78-1.29)	0.99
H5P	0.92 (0.74-1.14)	0.44	0.90 (0.73-1.12)	0.35
H6P	1.05 (0.82-1.36)	0.69	0.94 (0.71-1.25)	0.65
H7P	1.15 (0.88-1.49)	0.31	1.02 (0.75-1.38)	0.91

The analyses are based on 5151 participants (without pre-existing CVD), 323 CVD, 232 CHD and 87 stroke events.

CHD, coronary heart disease; CI, confidence interval; CVD, cardiovascular disease; H1P-H7P, high-density lipoprotein 1-7 particles (from smallest to largest); HDL-C, high-density lipoprotein cholesterol; HDL-P, high-density lipoprotein particles; HR, hazard ratio; NA, not applicable; SD, standard deviation

Model 1: adjustment for age, sex, smoking status, history of type 2 diabetes, systolic blood pressure, total cholesterol, triglycerides, body mass index, glucose, estimated glomerular filtration rate, high-sensitivity C-reactive protein, albumin, gamma glutamyltransferase, and alanine aminotransferase

Model 2: Model 1 plus HDL-C

Table S4. Associations of HDL particles and subspecies with cardiovascular outcomes (with further adjustment for HDL-P)

HDL exposures<br>Per SD increase	Model 1		Model 2	
	**HR (95% CI)**	***p*-value**	**HR (95% CI)**	***p*-value**
**Cardiovascular disease**				
HDL-C	0.82 (0.71-0.95)	0.009	0.83 (0.69-0.99)	0.041
HDL-P	0.90 (0.80-1.02)	0.098	NA	NA
Large HDL	0.92 (0.78-1.08)	0.30	0.96 (0.81-1.12)	0.57
Medium HDL	0.88 (0.78-1.01)	0.070	0.91 (0.79-1.05)	0.21
Small HDL	1.01 (0.89-1.15)	0.88	1.12 (0.95-1.31)	0.18
HDL size	0.89 (0.76-1.04)	0.14	0.91 (0.78-1.07)	0.24
H1P	1.02 (0.91-1.14)	0.74	1.04 (0.93-1.17)	0.47
H2P	0.99 (0.87-1.13)	0.92	1.06 (0.92-1.22)	0.43
H3P	0.92 (0.81-1.04)	0.19	0.95 (0.83-1.08)	0.40
H4P	0.91 (0.80-1.04)	0.19	0.93 (0.81-1.07)	0.33
H5P	0.94 (0.83-1.05)	0.27	0.94 (0.84-1.06)	0.33
H6P	0.93 (0.82-1.06)	0.28	0.95 (0.83-1.09)	0.46
H7P	0.94 (0.83-1.06)	0.30	0.96 (0.84-1.09)	0.53
**Coronary heart disease**				
HDL-C	0.70 (0.58-0.85)	<0.001	0.69 (0.55-0.88)	0.002
HDL-P	0.86 (0.74-0.99)	0.040	NA	NA
Large HDL	0.83 (0.68-1.02)	0.081	0.88 (0.71-1.08)	0.22
Medium HDL	0.82 (0.69-0.96)	0.014	0.85 (0.71-1.01)	0.064
Small HDL	1.04 (0.89-1.21)	0.62	1.24 (1.02-1.51)	0.032
HDL size	0.78 (0.64-0.94)	0.011	0.80 (0.66-0.97)	0.027
H1P	0.99 (0.87-1.13)	0.91	1.02 (0.90-1.17)	0.72
H2P	1.05 (0.91-1.23)	0.49	1.20 (1.00-1.42)	0.045
H3P	0.89 (0.77-1.03)	0.12	0.93 (0.80-1.08)	0.34
H4P	0.81 (0.68-0.97)	0.021	0.84 (0.70-1.00)	0.050
H5P	0.96 (0.84-1.11)	0.59	0.97 (0.85-1.12)	0.71
H6P	0.91 (0.79-1.06)	0.24	0.94 (0.81-1.10)	0.46
H7P	0.88 (0.77-1.02)	0.095	0.92 (0.79-1.07)	0.27
**Stroke**				
HDL-C	1.22 (0.97-1.54)	0.094	1.24 (0.92-1.67)	0.15
HDL-P	1.11 (0.88-1.39)	0.39	NA	NA
Large HDL	1.21 (0.94-1.55)	0.14	1.19 (0.91-1.54)	0.21
Medium HDL	1.18 (0.94-1.48)	0.16	1.16 (0.90-1.50)	0.26
Small HDL	0.90 (0.70-1.15)	0.38	0.80 (0.60-1.06)	0.13
HDL size	1.27 (0.98-1.65)	0.071	1.26 (0.96-1.64)	0.096
H1P	0.97 (0.78-1.21)	0.80	0.95 (0.76-1.19)	0.66
H2P	0.91 (0.71-1.15)	0.43	0.84 (0.65-1.10)	0.21
H3P	1.14 (0.91-1.42)	0.26	1.11 (0.88-1.41)	0.38
H4P	1.10 (0.88-1.37)	0.41	1.07 (0.85-1.36)	0.55
H5P	0.92 (0.74-1.14)	0.44	0.91 (0.73-1.13)	0.38
H6P	1.05 (0.82-1.36)	0.69	1.02 (0.78-1.34)	0.86
H7P	1.15 (0.88-1.49)	0.31	1.11 (0.85-1.46)	0.43

The analyses are based on 5151 participants (without pre-existing CVD), 323 CVD, 232 CHD and 87 stroke events.
CHD, coronary heart disease; CI, confidence interval; CVD, cardiovascular disease; H1P-H7P, high-density lipoprotein 1-7 particles (from smallest to largest); HDL-C, high-density lipoprotein cholesterol; HDL-P, high-density lipoprotein particles; HR, hazard ratio; NA, not applicable; SD, standard deviation
Model 1: Adjustment for age, sex, smoking status, history of type 2 diabetes, systolic blood pressure, total cholesterol, triglycerides, body mass index, glucose, estimated glomerular filtration rate, high-sensitivity C-reactive protein, albumin, gamma glutamyltransferase, and alanine aminotransferase
Model 2: Model 1 plus HDL-C

Table S5. Associations of HDL particles and subspecies with CVD according to alcohol consumption categories

HDL exposures Per SD increase	Alcohol consumption, g/day 0/rarely HR (95% CI)	*p*-value	0.1-10 HR (95% CI)	*p*-value	10-30 HR (95% CI)	*p*-value	>30 HR (95% CI)	*p*-value	*p*-value for interaction
HDL-C	0.89 (0.70-1.15)	0.38	0.75 (0.60-0.95)	0.015	0.93 (0.72-1.19)	0.56	0.79 (0.49-1.27)	0.32	0.57
HDL-P	0.86 (0.68-1.08)	0.19	0.88 (0.73-1.06)	0.19	0.97 (0.78-1.21)	0.79	1.18 (0.75-1.86)	0.47	0.56
Large HDL	1.07 (0.84-1.36)	0.60	0.89 (0.70-1.13)	0.35	0.92 (0.69-1.23)	0.58	0.56 (0.26-1.20)	0.14	0.36
Medium HDL	0.99 (0.78-1.25)	0.90	0.81 (0.66-1.01)	0.059	0.91 (0.71-1.16)	0.43	0.97 (0.66-1.42)	0.87	0.65
Small HDL	0.84 (0.67-1.06)	0.15	1.04 (0.86-1.27)	0.66	1.08 (0.85-1.38)	0.54	1.46 (0.91-2.36)	0.12	0.16
HDL size	1.01 (0.79-1.29)	0.92	0.87 (0.70-1.08)	0.19	0.90 (0.69-1.18)	0.46	0.72 (0.42-1.22)	0.22	0.59
H1P	0.99 (0.81-1.20)	0.89	1.03 (0.87-1.22)	0.72	1.03 (0.82-1.29)	0.82	0.95 (0.60-1.49)	0.82	0.97
H2P	0.86 (0.69-1.06)	0.16	1.02 (0.84-1.24)	0.83	1.07 (0.84-1.36)	0.59	1.59 (1.01-2.50)	0.045	0.085
H3P	1.01 (0.81-1.25)	0.93	0.87 (0.71-1.06)	0.16	0.94 (0.74-1.20)	0.63	0.92 (0.62-1.38)	0.70	0.78
H4P	0.97 (0.76-1.24)	0.79	0.87 (0.70-1.07)	0.19	0.91 (0.71-1.17)	0.47	1.09 (0.73-1.63)	0.69	0.76
H5P	0.94 (0.77-1.15)	0.54	1.16 (0.97-1.37)	0.095	1.13 (0.90-1.42)	0.30	1.09 (0.58-2.02)	0.81	0.45
H6P	1.11 (0.89-1.38)	0.37	0.82 (0.64-1.07)	0.14	0.78 (0.55-1.10)	0.16	0.29 (0.09-0.96)	0.042	0.042
H7P	1.03 (0.78-1.37)	0.81	0.84 (0.65-1.08)	0.18	1.01 (0.80-1.28)	0.92	0.88 (0.55-1.40)	0.58	0.62

The analyses are based on 5151 participants (without pre-existing CVD) and 323 CVD events.

CI, confidence interval; H1P-H7P, high-density lipoprotein 1-7 particles (from smallest to largest); HDL-C, high-density lipoprotein cholesterol; HDL-P, high-density lipoprotein particles; HR, hazard ratio

Small HDL: H1P and H2P; medium HDL: H3P-H4P; large HDL: H5P-H7P

Analyses were adjusted for age, sex, smoking status, history of type 2 diabetes, systolic blood pressure, total cholesterol, triglycerides, body mass index, glucose, estimated glomerular filtration rate (as calculated using the Chronic Kidney Disease Epidemiology Collaboration combined creatinine– cystatin C equation) high-sensitivity C-reactive protein, albumin, gamma glutamyltransferase, and alanine aminotransferase

Table S6. STROBE Checklist

Section/Topic	Item #	Recommendation	Reported on page #
**Title and abstract**	1	(*a*) Indicate the study's design with a commonly used term in the title or the abstract	Page 1
		(*b*) Provide in the abstract an informative and balanced summary of what was done and what was found	Page 2
**Introduction**			
Background/rationale	2	Explain the scientific background and rationale for the investigation being reported	Page 4
Objectives	3	State specific objectives, including any prespecified hypotheses	Page 4
**Methods**			
Study design	4	Present key elements of study design early in the paper	Study design and population
Setting	5	Describe the setting, locations, and relevant dates, including periods of recruitment, exposure, follow-up, and data collection	Study design and population
Participants	6	(*a*) Give the eligibility criteria, and the sources and methods of selection of participants. Describe methods of follow-up	Study design and population
		(*b*) For matched studies, give matching criteria and number of exposed and unexposed	Not applicable
Variables	7	Clearly define all outcomes, exposures, predictors, potential confounders, and effect modifiers. Give diagnostic criteria, if applicable	Assessment of exposures and other risk markers
Data sources/ measurement	8*	For each variable of interest, give sources of data and details of methods of assessment (measurement). Describe comparability of assessment methods if there is more than one group	Assessment of exposures and other risk markers
Bias	9	Describe any efforts to address potential sources of bias	Statistical analyses
Study size	10	Explain how the study size was arrived at	Statistical analyses
Quantitative variables	11	Explain how quantitative variables were handled in the analyses. If applicable, describe which groupings were chosen and why	Statistical analyses
Statistical methods	12	(*a*) Describe all statistical methods, including those used to control for confounding	Statistical analyses
		(*b*) Describe any methods used to examine subgroups and interactions	Statistical analyses
		(*c*) Explain how missing data were addressed	Not applicable
		(*d*) If applicable, explain how loss to follow-up was addressed	Not applicable
		(*e*) Describe any sensitivity analyses	Statistical analyses
**Results**			

Participants	13*	(a) Report numbers of individuals at each stage of study—eg numbers potentially eligible, examined for eligibility, confirmed eligible, included in the study, completing follow-up, and analysed	Study design and population
		(b) Give reasons for non-participation at each stage	Study design and population
		(c) Consider use of a flow diagram	Study design and population
Descriptive data	14*	(a) Give characteristics of study participants (eg demographic, clinical, social) and information on exposures and potential confounders	Results; Table 1
		(b) Indicate number of participants with missing data for each variable of interest	
		(c) Summarise follow-up time (eg, average and total amount)	Results
Outcome data	15*	Report numbers of outcome events or summary measures over time	Results
Main results	16	(*a*) Give unadjusted estimates and, if applicable, confounder-adjusted estimates and their precision (eg, 95% confidence interval). Make clear which confounders were adjusted for and why they were included	Results; Figure 1; Tables 2-5; Tables S1-S5
		(*b*) Report category boundaries when continuous variables were categorized	Results; Tables 2-5. Tables S1-S5
		(*c*) If relevant, consider translating estimates of relative risk into absolute risk for a meaningful time period	
Other analyses	17	Report other analyses done—eg analyses of subgroups and interactions, and sensitivity analyses	Results; Tables S1-S5
**Discussion**			
Key results	18	Summarise key results with reference to study objectives	Discussion
**Limitations**			
Interpretation	20	Give a cautious overall interpretation of results considering objectives, limitations, multiplicity of analyses, results from similar studies, and other relevant evidence	Discussion
Generalisability	21	Discuss the generalisability (external validity) of the study results	Discussion
**Other information**			
Funding	22	Give the source of funding and the role of the funders for the present study and, if applicable, for the original study on which the present article is based	Title page

Figure S1. Derivation of the analytic sample

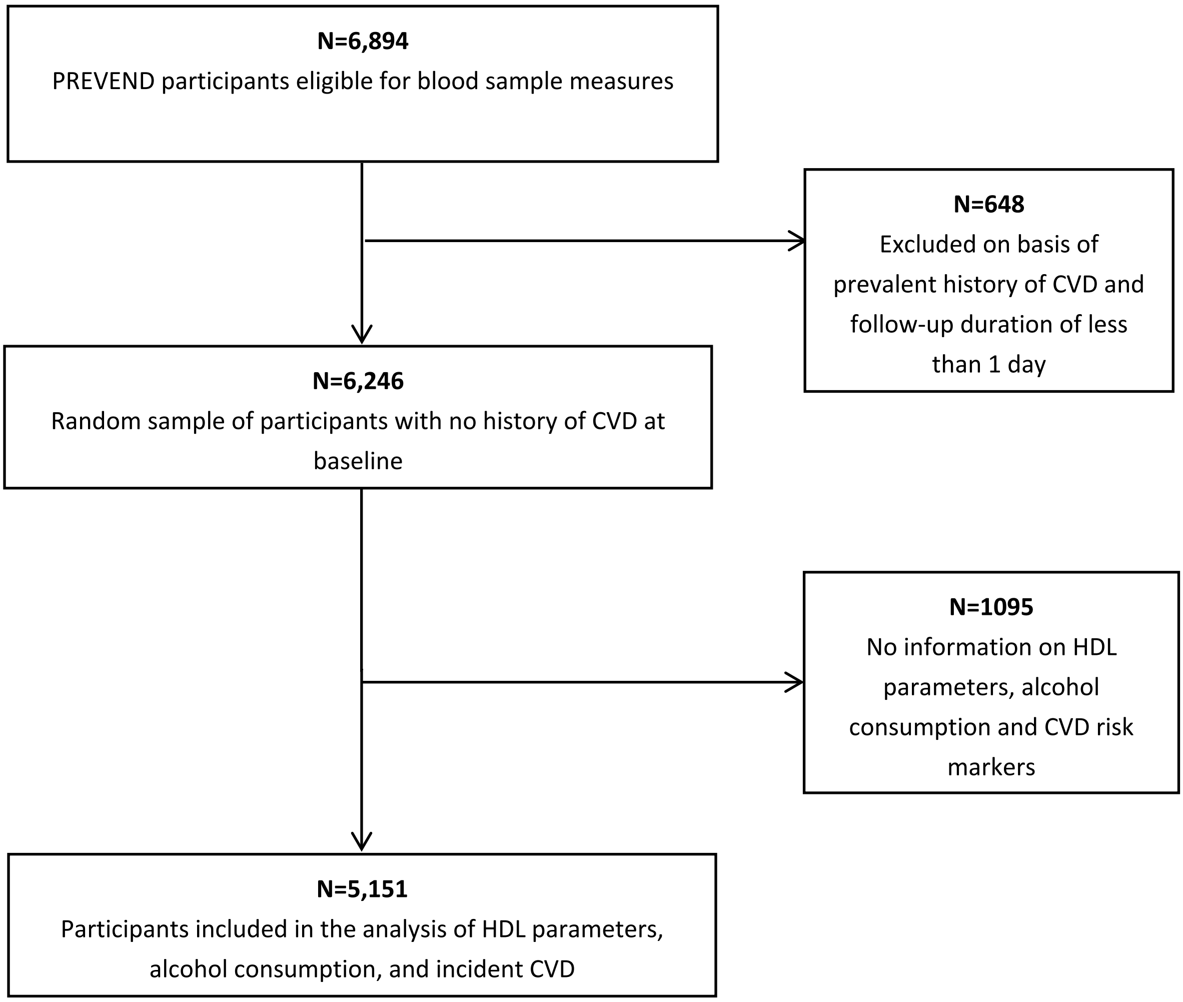

CVD, cardiovascular disease; HDL, high-density lipoproteins
